# Editorial: Methods in biofilms: 2022

**DOI:** 10.3389/fcimb.2023.1264849

**Published:** 2023-08-04

**Authors:** Romain Briandet, Nuno Cerca

**Affiliations:** ^1^ Université Paris-Saclay, INRAE, AgroParisTech, Micalis Institute, Jouy-en-Josas, France; ^2^ Laboratory of Research in Biofilms Rosário Oliveira (LIBRO), Centre of Biological Engineering (CEB), University of Minho, Braga, Portugal

**Keywords:** biofilm, methods, antimicrobial, emerging properties, health, industry, environment

Biofilms, complex communities of microorganisms adhering to surfaces, play a significant role in various fields, including healthcare, industry, and the environment (Flemming et al., 2016). As our understanding of biofilms expands, so does the need for advanced research methods and techniques to study their formation, composition, and physiological characteristics (Azeredo et al., 2017). This Research Topic aims to present a captivating collection of articles that highlight novel methodologies in biofilm research.

Biofilms present researchers with a range of challenges and opportunities. The articles in this Research Topic address these challenges by focusing on the development and adaptation of cutting-edge methods for studying biofilms. By bridging the gap between different scientific disciplines, these contributions provide a comprehensive view of biofilm research. From investigating the dynamics of antibiotic resistance selection using microfluidic chips to the specific detection of multiple pathogens in co-infection and mixed biofilms, these articles offer valuable insights into the complex nature of biofilm formation and interactions.

The Research Topic features articles that showcase innovative methodologies to study various aspects of biofilms. For instance, an article introduces a microfluidic chip for real-time monitoring of antibiotic resistance selection in bacterial biofilms (Tang et al.). This technology provides a valuable tool for understanding the dynamics of antibiotic resistance emergence and selection. Another article describes the development of a TaqMan duplex real-time PCR method for simultaneous detection of *Streptococcus suis* and *Actinobacillus pleuropneumoniae* in co-infection and mixed biofilms (Yi et al.). This method offers enhanced specificity and sensitivity for accurate quantification of these pathogens, facilitating disease prevention and control. Additionally, the Research Topic includes an upgraded repository of antimicrobial peptides (AMPs) for biofilm studies. This resource, B-AMP v2.0, provides a comprehensive collection of biofilm protein targets and AMPs, facilitating research into specific biofilm targets and anti-biofilm strategies (Ravichandran et al.). Furthermore, the utilization of fluorescence *in situ* hybridization (FISH) techniques is explored in another article, showcasing its applications for visualizing and quantifying microorganisms, genes, and metabolites within biofilms (Barbosa et al.). These advancements in FISH-based techniques offer valuable insights into biofilm structure and function. The innovative methodologies presented in this Research Topic contribute to our understanding of biofilm-related infections and provide avenues for the development of targeted interventions. Accurately quantifying bacterial burden in biofilms is crucial for studying their pathogenesis, and an article introduces a novel qPCR standard that improves the accuracy of quantification in vaginal microbial communities, including those associated with bacterial vaginosis (Elnaggar et al.). This advancement enhances our ability to study the role of biofilms in this prevalent vaginal dysbiosis, paving the way for improved diagnostics and treatment strategies.

In conclusion, the articles in this Research Topic underscore the significance of innovative methodologies in advancing our knowledge of biofilms. From microfluidic chips and molecular detection techniques to AMP repositories and FISH-based imaging, these contributions offer valuable tools for studying biofilm formation, structure, and function. By presenting these advancements, we aim to inspire further exploration, collaboration, and interdisciplinary research in the field of biofilm studies. Through continuous innovation, we can unlock the mysteries of biofilms, leading to improved strategies for managing biofilm-related challenges in various domains.

## Author contributions

RB: Conceptualization, Writing – original draft, Writing – review & editing. NC: Conceptualization, Writing – original draft, Writing – review & editing.
